# Effect of Vitamins C and E on Endothelial Function in Type 1 Diabetes Mellitus

**DOI:** 10.1155/2016/3271293

**Published:** 2015-12-10

**Authors:** Rachel-Marie Cazeau, Hong Huang, John A. Bauer, Robert P. Hoffman

**Affiliations:** ^1^Section of Endocrinology, Metabolism and Diabetes, Nationwide Children's Hospital, 700 Children's Drive, Columbus, OH 43205, USA; ^2^Division of Pediatric Endocrinology, Metabolism and Diabetes, Department of Pediatrics, The Clinical Research Center, The Ohio State University College of Medicine and Public Health, Columbus, OH 43205, USA; ^3^Department of Pediatrics, University of Kentucky, Lexington, KY 40506, USA; ^4^The Research Institute, Nationwide Children's Hospital, Columbus, OH 43205, USA

## Abstract

*Background/Objectives*. Endothelial dysfunction due to hyperglycemia-induced oxidative damage is an important predictor of future cardiovascular risk in patients with type 1 diabetes mellitus (T1DM) and is present in adolescent T1DM. We hypothesized that combined treatment with the antioxidant vitamins C and E might improve endothelial function (EF) and other biochemical risk factors in adolescents with T1DM. *Subjects/Methods*. Open-label antioxidant supplementation was given for six weeks with endpoint measurements collected at baseline and study completion. Endpoints measured included EF and plasma measurements of biochemical endothelial risk. *Results*. Two males and 7 females were studied. Mean age was 12.9 ± 0.9 yrs; mean T1DM duration was 5.5 ± 2.5 yrs; mean BMI was 22.1 ± 3.8 kg/m^2^; and mean hemoglobin A1c was 9.3 ± 1.1%. No differences were found for EF, high sensitivity CRP, total antioxidant capacity, adiponectin, or endothelial progenitor cells (EPCs) between before and after combined vitamin C and E therapy. *Conclusions*. Our negative study results do not support previous findings of decreased oxidative damage, improved endothelial function, and increased vascular repair capacity with antioxidant therapy. Longer term studies may be needed to determine the effects, if any, of combined antioxidant therapy on EPCs, EF, and markers of micro- and macrovascular complications in T1DM.

## 1. Introduction

Type 1 diabetes mellitus (T1DM) is a chronic medical condition requiring intensive treatment and optimal glycemic control in order to prevent and/or delay onset and/or progression of long term complications. Cardiovascular disease (CVD) is the major cause of death for patients with T1DM in the United States. The mortality and morbidity of CVD are strikingly increased in individuals with T1DM compared to their nondiabetic counterparts [1, 2] with a 6–13-fold higher risk of cardiovascular death [3]. Atherosclerosis, the major pathophysiologic precursor of cardiovascular disease and ultimate cause cardiovascular morbidity and mortality, begins in childhood and adolescence [4]. Endothelial dysfunction is an early marker of atherosclerosis that begins early in T1DM and is directly involved in diabetes-induced microvascular and macrovascular complications [1, 3, 5–9]. Although clinically manifested diabetes-related microvascular (e.g., retinopathy, nephropathy, and neuropathy) and macrovascular complications (e.g., cardiac disease and peripheral vascular disease) are rare during childhood or adolescence, it is evident that early potentially modifiable vascular functional and structural changes begin soon after diagnosis.

Endothelial dysfunction, vascular inflammation, build-up of lipids, cholesterol, calcium, and cellular debris within the intima of the vessel wall are characteristic features of atherosclerosis [10]. The development of atherosclerosis in T1DM begins in childhood and adolescence as a largely silent process as shown by increased intima-media thickness (IMT) of the carotids and aorta [11, 12], impaired endothelial function, and altered endothelial repair capacity. Impaired endothelial function and altered endothelial repair have been observed in T1DM. Previous studies have not only found reduced flow mediated vasodilation [11, 13, 14], a measure of endothelial function, but also decreased CD34+ CD133+ CD31+ circulating progenitor cells (CPCs) cells with increased CD34+ CD45− endothelial colony forming cells (ECFCs) [15], an indicator of altered endothelial repair, in children and adolescents with T1DM. Additionally, hyperglycemia-induced oxidative stress plays a pivotal pathophysiological role in impairing endothelial function in T1DM [6, 7]. Numerous studies have demonstrated that hyperglycemia not only increases free radicals but also impairs endogenous antioxidant defense in T1DM. Children with T1DM have evidence of increased oxidative stress in blood and reductions in various aspects of antioxidant defense, including reduced endogenous levels of vitamins C and E, glutathione, decreased levels of superoxide dismutase, total antioxidant capacity (TAC), increased levels of malondialdehyde, and oxidized LDL [16, 17].

While optimal glycemic control has been associated with improved cardiac and peripheral vascular function and the prevention of later vascular complications, discovering a means of reducing or eliminating these early vascular changes of CVD seen in childhood that is either independent of or augmenting optimal glycemic control would be a major medical breakthrough. Enhanced antioxidant intake (either via diet or supplementation) may be one such means of reducing the risk of diabetes-related microvascular and macrovascular complications later in life, increasing the lifespan and wellness, and reducing anxiety and frustration in patients with T1DM.

The objective of this study was to determine effects of antioxidant therapy, specifically vitamins C and E, on antioxidant capacity, inflammatory markers (CRP and IL6), and endothelial function, and define their interrelationships in adolescents with T1DM. We also set out to determine the effects of one month of combined antioxidant therapy on numbers of endothelial colony forming cells (ECFCs: CD34+ CD133+ CD45−) in adolescents with T1DM. We hypothesized that antioxidant vitamin therapy would decrease oxidative damage thereby improving endothelial function and increase vascular repair capacity in adolescent T1DM.

## 2. Materials and Methods

### 2.1. Subjects

Nine children and adolescents aged 8–15 years (mean age ± SD) with T1DM were included in this study. These subjects were recruited from Pediatric Diabetes Clinics Nationwide Children's Hospital in Columbus, Ohio. Informed consent was obtained from a parent or legal guardian and informed assent was obtained from the subject. The study was approved by the Institutional Review Board of Nationwide Children's Hospital. The initial diagnosis of T1DM will have been made clinically. They must have been started on insulin therapy immediately after diagnosis and never have received an oral hypoglycemic agent.

For inclusion, subjects were between 8 and 15 years of age with BMI ≤ 95% tile for age, pubertal stage of Tanner stages 2–4, BP ≤ 95% tile for age, on insulin therapy since diagnosis, fasting c-peptide <0.4 ng/mL, normal thyroid function tests, random urine albumin to creatinine ratio <0.02 mg albumin/mg creatinine, and creatinine ≤1.0 mg/dL. Subjects with BMI ≥ 95% tile, BP > 95% tile, Tanner 1 or 5 pubertal status, pregnancy, smoking, history of oral hypoglycemic use, acanthosis nigricans, fasting c-peptide ≥0.4 ng/mL, abnormal thyroid function tests, random urine albumin to creatinine ratio ≥0.02 mg albumin/mg creatinine, creatinine >1.0 mg/dL, or use of any medications other than insulin, levothyroxine with stable dosage, or oral contraceptives were excluded from the study.

### 2.2. Protocol

This was an open-label antioxidant supplementation for six weeks with endpoint measurements collected at baseline and study completion. The subjects were seen twice at the Clinical Research Center of the Clinical and Translational Study Center of The Ohio State University. The first visit occurred the morning after a minimum of 10-hour fasting. Subjects were instructed to take their usual insulin the day before their study visit and to take any morning basal insulin. Subjects using continuous insulin pump therapy were continued on their usual basal insulin rates. Morning rapid acting insulin injection or insulin pump boluses were held. Baseline blood samples were drawn for total plasma antioxidant capacity (TAOC), high-sensitivity C-reactive protein (hsCRP), and measurement of endothelial progenitor cells (EPCs). Female participants were evaluated during the first two weeks of their menstrual cycle in an attempt to minimize the possible effects of hormonal changes on endothelial function [18, 19]. Endothelial function was then measured as described below. After completion of the measurements subjects took their morning rapid acting insulin injection or insulin pump bolus and were given breakfast. The second study visit was identical to the first with measurement of all endpoints. Subjects were instructed to return all medications at visit two.

### 2.3. Endothelial Function Measurement

Endothelium dependent vasodilatory response was quantified as the mean postupper arm occlusion forearm vascular resistance (FVR) and the percent change in forearm vascular resistance from pre- to postocclusion. Forearm blood flow (FBF) was measured using strain gauge venous occlusion plethysmography using a Hokanson AI6 plethysmograph. Two minutes of baseline FBF was recorded after which the upper arm cuff was inflated to 200 mmHg pressure for 5 minutes to occlude flow. It was then released and FBF was measured for one minute. FVR was calculated by dividing mean arterial blood pressure by FBF. Arterial blood pressure was measured using an automated sphygmomanometer. This method of testing endothelial function assesses resistance vessel function. Results closely correlate with results from endothelial function assessed by intra-arterial acetylcholine infusion [20] and correlate well with the nitrite/nitrate ratio, an index of NO synthesis [21].

### 2.4. Vitamin Supplementation

Prior to discharge, subjects were started on combined vitamins C and E. We scaled our supplement dosing to account for subject size, as shown in Table 1; these doses are equivalent to a dose of 1 g of vitamin C and 400 IU of vitamin E per day in a 75 kg adult [16]. Subjects were instructed to take the medication once daily and were instructed to continue their routine diabetes care between visits.

### 2.5. Laboratory Assays

High-sensitivity C-reactive protein (hsCRP) and total plasma antioxidant capacity (TAOC), a measure of oxidative stress, were measured for each subject at their baseline fasting glucose level. TAOC is a nonspecific assay of antioxidant defense which measures the ability of constituents in plasma to absorb oxidation (BioVision Research Products, Mountain View, CA). Adiponectin was measured using a kit from R&D Systems Inc. Minneapolis, MN, Cat. Number 1065. ECFCs were measured using polychromatic flow cytometry methods. A 50 *μ*L volume anticoagulated peripheral blood was incubated with 50 *μ*L 3% BSA in PBS (without Ca++ and Mg++) at room temperature for 30 min. In dark, fluorescence labeled antibodies (2.5 *μ*L of each), PE-AC133, FITC-CD34, and PECy5-CD45, were added and incubated for 30 min at room temperature. FACS lysis buffer (450 *μ*L) was then added and incubated for 30 min at room temperature in dark. Samples were then analyzed on FACS Caliber flow cytometer, where total counts are >400,000 cells. Intra-assay variability from ~100 *μ*L whole blood was <5%.

### 2.6. Statistical Analysis

Data were analyzed using a paired *t*-test. Results are presented as mean ± SD.

## 3. Results

**Figure 1:**
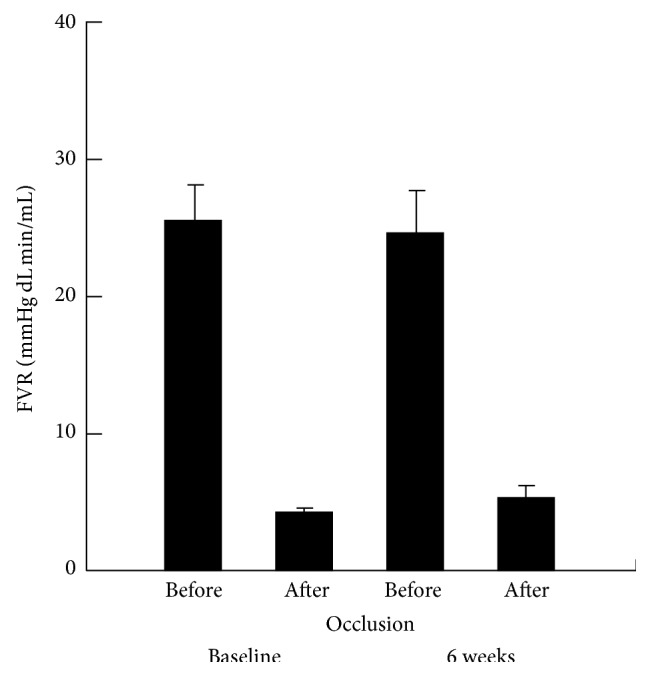
FVR response before and after occlusion before and after combined vitamin C and E therapy.

Two males and 7 females were studied. Mean age was 12.9 ± 0.9 years (range, 11.2 to 13.9 years): mean duration of diabetes was 5.5 ± 2.5 years (range, 0.2 to 9.5 years); mean BMI was 22.1 ± 3.8 kg/m^2^ (range 18.2 to 29.4 kg/m^2^); and mean hemoglobin A1 was 9.3 ± 1.1% (range, 8.2% to 11.3%). No differences were seen in the FVR response to occlusion between before and after combined vitamin C and E therapy (Figure 1). No differences were seen in hsCRP, TAOC, adiponectin, or EPC percent before or after vitamin C and E therapy (Table 2).

## 4. Discussion 

Oxidative stress plays a significant role in the chronic complications of insulin-dependent diabetes mellitus. Our study set out to determine the effects of antioxidant therapy on antioxidant capacity, inflammatory markers, endothelial function, and numbers of ECFCs, in adolescents with T1DM. The oxidative stress that impairs endothelial function in T1DM is induced by hyperglycemia [6, 7]. This hyperglycemia also impairs the endogenous antioxidant defense in T1DM. Children with T1DM have evidence of reduced levels of endogenous antioxidants vitamins C and E [16, 17]. Under normal conditions, vitamin E suppresses the propagation of lipid peroxidation and scavenges free radicals; and vitamin C with vitamin E inhibits hydroperoxide formation; metal complexing agents, such as penicillamine, bind transition metals involved in some reactions in lipid peroxidation and inhibit Fenton- and Haber-Weiss-type reactions (i.e., generating hydroxyl radicals (OH) from hydrogen peroxide (H_2_O_2_) and superoxide (O_2_^−^)) [22–25]. We hypothesized that antioxidant vitamin therapy would decrease oxidative damage thereby improving endothelial function and vascular repair capacity in adolescent T1DM. This hypothesis proved to be incorrect as we found no changes in postocclusive FVR, hsCRP, TAC, adiponectin, or ECFCs.

Low vitamin C levels in type 1 diabetes are associated with increased transcapillary albumin escape [26] and increased atherosclerotic damage as evidenced by a negative relationship to IMT in children [27]. Previous studies with ascorbic acid (vitamin C) had suggested potential benefit. Antioxidant treatment with acute intravenous ascorbic acid or 10 days of oral ascorbic acid blocks the dilatory effect of hyperglycemia on endothelial function during hyperglycemic clamp [28] or oral glucose tolerance testing [29] in healthy adults. A 12-hour ascorbic acid infusion restores endothelial function to normal levels in adults with recent onset or intermediate duration, well-controlled type 1 diabetes [30, 31]. In patients with poorly controlled type 1 diabetes ascorbic acid infusion, alone, only partially restores endothelial function. Acute ascorbic acid infusion blocks the acute effects of hyperglycemia in adolescents with type 1 diabetes [32]. Therapeutically, vitamin C decreases transcapillary albumin escape [26] and urinary albumin excretion [33] in adults with T1DM. A previous study by Varvaroksa et al. found in children with T1DM the combination of vitamins C and E decreased hemoglobin A1c and glycated protein levels and increased superoxide dismutase and reduced glutathione [16]. However, based on our results, combined vitamin C and E antioxidant intake via supplementation did not improve endothelial function, ECFCs, or other nontraditional risk factors.

Limitations of our study may include the short duration of vitamin C and E supplementation for 6 weeks which may have been inadequate to significantly decrease oxidative damage, improve endothelial function, and increase vascular repair capacity. The strategy (e.g., dosage and duration) was chosen based on studies in adults, in many varying trials, which showed that vitamin C (at doses 1 g/day or less) and vitamin E (at doses of 400 IU/day or less) were consistently shown to be safe and in many specific settings relevant to this study to have efficacy in reducing oxidative stress in vivo and/or affecting markers of disease. Our study utilized these two supplements in combination, primarily since we do not know if water soluble or fat soluble strategies are best in this setting and since it is clear that these and other small molecule antioxidants are compartmentalized in vivo and interact to affect cellular redox status [34]. Also other studies have suggested that lower doses of multiple antioxidants may indeed be superior (and safer than) to mega doses of one constituent [35–37]. Also our small sample size may not have been large enough to demonstrate significant differences. The lack of a normal control group is also another limitation to this study. The differences seen were small and even with a sample size large enough to find statistical significance are unlikely to be of any clinical significance.

## 5. Conclusions

Our negative study results do not support previous findings of decreased oxidative damage, improved endothelial function, and increased vascular repair capacity with antioxidant therapy. Further research into other means of reducing or eliminating vascular changes of CVD seen in childhood that is either independent of or augmenting optimal glycemic control is indeed warranted. Ideally these studies would begin at time of diagnosis of T1DM before the development of diabetic complications. Longer term studies are necessary to determine the effects, if any, of combined antioxidant therapy on ECFCs, endothelial function, and markers of microvascular and macrovascular complications in T1DM.

## Figures and Tables

**Table 1:** Antioxidant dosage.

Dosing body weight	Daily vitamin C dose (mg)	Daily vitamin E dose (IU)
<30 kg	250 mg	100 IU
30–60 kg	500 mg	200 IU
>60 kg	750 mg	300 IU

**Table 2:** Biochemical markers and ECFCs at baseline and after 6 weeks of vitamin C and E therapy in adolescents with type 1 diabetes.

	hsCRP (ng/mL)	TAOC (Trolox equivalent/L)	Adiponectin (ng/mL)	ECFC (%)
Baseline	0.63 ± 0.85	95 ± 6	3970 ± 1150	0.027 ± 0.015
Vitamins C and E	0.74 ± 0.88	102 ± 12	4330 ± 1280	0.032 ± 0.037

## Abbreviations

BSA: Body surface area
ECFC: Endothelial colony forming cells
EPC: Endothelial progenitor cells
FBF: Forearm blood flow
FVR: Forearm vascular resistance
hsCRP: High-sensitivity C-reactive protein
TAOC: Total plasma antioxidant capacity
T1DM: Type 1 diabetes mellitus.

## References

[1] Donaghue K. C., Wadwa R. P., Dimeglio L. A. (2014). Microvascular and macrovascular complications in children and adolescents. *Pediatric Diabetes*.

[2] Laing S. P., Swerdlow A. J., Slater S. D. (2003). Mortality from heart disease in a cohort of 23,000 patients with insulin-treated diabetes. *Diabetologia*.

[3] Giannini C., Mohn A., Chiarelli F., Kelnar C. J. H. (2011). Macrovascular angiopathy in children and adolescents with type 1 diabetes. *Diabetes/Metabolism Research and Reviews*.

[4] McGill H. C., McMahan C. A., Herderick E. E., Malcom G. T., Tracy R. E., Strong J. P. (2000). Origin of atherosclerosis in childhood and adolescence. *The American Journal of Clinical Nutrition*.

[5] Ross R. (1999). Atherosclerosis—an inflammatory disease. *The New England Journal of Medicine*.

[6] Cai H., Harrison D. G. (2000). Endothelial dysfunction in cardiovascular diseases: the role of oxidant stress. *Circulation Research*.

[7] Stehouwer C. D. A., Lambert J., Donker A. J. M., van Hinsbergh V. W. M. (1997). Endothelial dysfunction and pathogenesis of diabetic angiopathy. *Cardiovascular Research*.

[8] Berenson G. S., Srinivasan S. R., Nicklas T. A. (1998). Atherosclerosis: a nutritional disease of childhood. *The American Journal of Cardiology*.

[9] Furumoto T., Saito N., Dong J., Mikami T., Fujii S., Kitabatake A. (2002). Association of cardiovascular risk factors and endothelial dysfunction in Japanese hypertensive patients: implications for early atherosclerosis. *Hypertension Research*.

[10] Tousoulis D., Kampoli A.-M., Papageorgiou N. (2011). Pathophysiology of atherosclerosis: the role of inflammation. *Current Pharmaceutical Design*.

[11] Järvisalo M. J., Raitakari M., Toikka J. O. (2004). Endothelial dysfunction and increased arterial intima-media thickness in children with type 1 diabetes. *Circulation*.

[12] Harrington J., Peña A. S., Gent R., Hirte C., Couper J. (2010). Aortic intima media thickness is an early marker of atherosclerosis in children with type 1 diabetes mellitus. *The Journal of Pediatrics*.

[13] Donaghue K. C., Robinson J., McCredie R., Fung A., Silink M., Celermajer D. S. (1997). Large vessel dysfunction in diabetic adolescents and its relationship to small vessel complications. *Journal of Pediatric Endocrinology and Metabolism*.

[14] Wiltshire E. J., Gent R., Hirte C., Pena A., Thomas D. W., Couper J. J. (2002). Endothelial dysfunction relates to folate status in children and adolescents with type 1 diabetes. *Diabetes*.

[15] DiMeglio L. A., Tosh A., Saha C. (2010). Endothelial abnormalities in adolescents with type 1 diabetes: a biomarker for vascular sequelae?. *Journal of Pediatrics*.

[16] Varvařovská J., Racek J., Štětina R. (2004). Aspects of oxidative stress in children with type 1 diabetes mellitus. *Biomedicine & Pharmacotherapy*.

[17] Wittenstein B., Klein M., Finckh B., Ullrich K., Kohlschütter A. (2002). Plasma antioxidants in pediatric patients with glycogen storage disease, diabetes mellitus, and hypercholesterolemia. *Free Radical Biology and Medicine*.

[18] Hashimoto M., Akishita M., Eto M. (1995). Modulation of endothelium-dependent flow-mediated dilatation of the brachial artery by sex and menstrual cycle. *Circulation*.

[19] Williams M. R. I., Westerman R. A., Kingwell B. A. (2001). Variations in endothelial function and arterial compliance during the menstrual cycle. *Journal of Clinical Endocrinology and Metabolism*.

[20] Higashi Y., Yoshizumi M. (2003). New methods to evaluate endothelial function: method for assessing endothelial function in humans using a strain-gauge plethysmography: nitric oxide-dependent and -independent vasodilation. *Journal of Pharmacological Sciences*.

[21] Sanada M., Higashi Y., Nakagawa K. (2002). Hormone replacement effects on endothelial function measured in the forearm resistance artery in normocholesterolemic and hypercholesterolemic postmenopausal women. *Journal of Clinical Endocrinology and Metabolism*.

[22] Maritim A. C., Sanders R. A., Watkins J. B. (2003). Diabetes, oxidative stress, and antioxidants: a review. *Journal of Biochemical and Molecular Toxicology*.

[23] Feher J., Cosmos G., Vereckei A. (1987). *Free Radical Reactions in Medicine*.

[24] Laight D. W., Carrier M. J., Änggård E. E. (2000). Antioxidants, diabetes and endothelial dysfunction. *Cardiovascular Research*.

[25] Chow C. K. (1991). Vitamin E and oxidative stress. *Free Radical Biology and Medicine*.

[26] Juhl B., Klein F., Christiansen J. S. (2004). Vitamin C treatment reduces transcapillary escape rate of albumin in type 1 diabetes. *European Journal of Internal Medicine*.

[27] Odermarsky M., Lykkesfeldt J., Liuba P. (2009). Poor vitamin C status is associated with increased carotid intima-media thickness, decreased microvascular function, and delayed myocardial repolarization in young patients with type 1 diabetes. *The American Journal of Clinical Nutrition*.

[28] Mullan B. A., Ennis C. N., Fee H. J. P., Young I. S., McCance D. R. (2005). Pretreatment with intravenous ascorbic acid preserves endothelial function during acute hyperglycaemia (R1). *Clinical and Experimental Pharmacology and Physiology*.

[29] De Marchi S., Prior M., Rigoni A., Zecchetto S., Rulfo F., Arosio E. (2012). Ascorbic acid prevents vascular dysfunction induced by oral glucose load in healthy subjects. *European Journal of Internal Medicine*.

[30] Ceriello A., Esposito K., Ihnat M., Thorpe J., Giugliano D. (2009). Long-term glycemic control influences the long-lasting effect of hyperglycemia on endothelial function in type 1 diabetes. *Journal of Clinical Endocrinology and Metabolism*.

[31] Ceriello A., Piconi L., Esposito K., Giugliano D. (2007). Telmisartan shows an equivalent effect of vitamin C in further improving endothelial dysfunction after glycemia normalization in type 1 diabetes. *Diabetes Care*.

[32] Hoffman R. P., Dye A. S., Bauer J. A. (2012). Ascorbic acid blocks hyperglycemic impairment of endothelial function in adolescents with type 1 diabetes. *Pediatric Diabetes*.

[33] McAuliffe A. V., Brooks B. A., Fisher E. J., Molyneaux L. M., Yue D. K. (1998). Administration of ascorbic acid and an aldose reductase inhibitor (tolrestat) in diabetes: effect on urinary albumin excretion. *Nephron*.

[34] May J. M., Qu Z.-C., Neel D. R., Li X. (2003). Recycling of vitamin C from its oxidized forms by human endothelial cells. *Biochimica et Biophysica Acta*.

[35] Miller E. R., Pastor-Barriuso R., Dalal D., Riemersma R. A., Appel L. J., Guallar E. (2005). Meta-analysis: high-dosage vitamin E supplementation may increase all-cause mortality. *Annals of Internal Medicine*.

[36] Padayatty S. J., Katz A., Wang Y. (2003). Vitamin C as an antioxidant: Evaluation of its role in disease prevention. *Journal of the American College of Nutrition*.

[37] Rimm E. B., Stampfer M. J., Ascherio A., Giovannucci E., Colditz G. A., Willett W. C. (1993). Vitamin E consumption and the risk of coronary heart disease in men. *The New England Journal of Medicine*.

